# Influence of Calomel on Digestion

**Published:** 1883-11

**Authors:** 


THE INFLUENCE OF CLAOMEL ON DIGESTION.

Dr. Vassillieff has found, from experiment, that the presence of calomel, at least up to the amount of five grammes, in the alimentary canal, does not interfere with the gastric juice, nor affect the triple influence of the pancreatic fluid on albumen, fat and starch ; on mixing the latter fluid with fibrin and calomel, the formation of certain products, idol, etc., always appearing as a result of prolonged digestion under normal circumstances, is prevented. The gases generated in the process of pancreatic digestion contain none of the usual products of fermentation and decomposition when calomel is present : sulphuretted hydrogen and pure hydrogen are absent, carbonic acid is diminished to from two to ten per cent.; whilst, under natural circumstances, from fourteen to 54 per cent. is found in the gases evolved by the action of the pancreatic fluid. In fact, calomel prevents all other changes in nutritious substances, save those produced entirely by the digestive secretions, decomposition and retrogressive processes in albumens being entirely checked. Calomel also prevents butyric acid fermentation, as Vassilieff found by experiments on cheese. The action of calomel readily explains the cause of the green color of fæces passed by patients to whom that drug has been administered. Hoppe-Seyler rightly attributed this coloration to the presence of unaltered bile. Now, under normal conditions, bilirubin and biliverdin are changed by a process of decomposition into hydrobilirubin, and thus become no longer recognizable in the excretion : but this process is arrested by calomel and the coloring agents, unaltered, give the fæces their peculiar bright green hue.

These researches are described at length by Dr. Vassilieff in the *Zeitschrift für Physiologische Chemic*, vol. vi, page 112. He has found that this action of calomel is due to its power over the micro-organisms intimately associated with the process of decomposition which takes place in food during digestion. The drug prevents the development of micro-organisms in the digestive fluids, and also destroys any bacteria and micrococci already developed. This fact was first proved by artificial digestion. Vassilieff then made a series of experiments to find whether calomel had the same influence in natural digestion. Thirty grains of calomel were administered to a dog, in two doses, and the animal was killed a few hours later. Under all precautions, the contents of the intestines were then carefully analyzed. Neither idol nor phenol could be found ; and it will not be forgotten by those who study contemporaneous physiological research, that other agents—such as salicylic acid—prevent the formation of idol ; and that pancreatic mixtures, formed from natural pancreatic juice, or infusions of pancreatic glandular tissue, undergo septic changes with very great rapidity in spite of all precautions. None of these chenges, nor any formation of idol, occurred in the food taken by dogs to which Vassilieff administered calomel. On the other hand, leucin and tyrosin were found in abundance. Under natural circumstances these products of pancreatic digestion are so rapidly decomposed that they cannot be detected in semi-digested food. Hence calomel has no influence on the action of digestive fluids, but entirely prevents those true retrogressive and putrefactive changes whereby the highly unstable products of these fluids are rapidly decomposed, and micro-organisms quickly developed in great numbers. When calomel enters the alimentary canal, leucin, tyrosin, bilirubin, and other substances, remain unchanged, and bacteria are checked and killed.—*British Medical Journal.*

